# Glutamine Metabolism in Cardiac Hypertrophy

**DOI:** 10.31083/RCM51889

**Published:** 2026-09-18

**Authors:** Zhi-Guo Hu, Yao Yao, Yan Gong, Yu-Ting Yang

**Affiliations:** ^1^Department of Critical Care Medicine, Medical Center of Anesthesiology and Pain, The First Affiliated Hospital, Jiangxi Medical College, Nanchang University, 330006 Nanchang, Jiangxi, China

**Keywords:** cardiac hypertrophy, glutamine metabolism, metabolic reprogramming, metabolic enzyme, transporter

## Abstract

Cardiac hypertrophy represents a crucial adaptive response of the heart to various physiological and pathological stimuli, with metabolic reprogramming serving as the core mechanism driving this process. In recent years, the role of glutamine metabolism in cardiac hypertrophy has garnered increasing attention. This review systematically elucidates the similarities and differences in glutamine metabolism between physiological and pathological cardiac hypertrophy, with a focus on the dual role of glutamine metabolism in regulating cardiomyocyte hypertrophy: it has a protective effect by maintaining energy metabolism and reducing oxidative stress, while simultaneously contributing to the hypertrophic process by promoting anabolic metabolism and exacerbating metabolic remodeling. The subsequent analysis delved into the specific mechanisms by which glutamine metabolism regulates cardiomyocyte hypertrophy, focusing on the functions of key metabolic enzymes and transporters that collectively govern glutamine flux and its downstream signaling pathways. Furthermore, we explore the intricate interplay between glutamine metabolism and lipid metabolism, which plays a pivotal role in cardiac energy homeostasis and hypertrophic development. Finally, we discuss the potential of targeting glutamine metabolism as a therapeutic strategy, highlighting the novel therapeutic prospects of glutamine supplementation in cardiac diseases. In summary, as a key component of metabolic reprogramming in cardiomyocyte hypertrophy, glutamine metabolism offers deeper insights into the pathophysiological mechanisms of cardiac hypertrophy. Future interventions targeting the glutamine metabolic pathway could prevent and treat pathological cardiac hypertrophy.

## 1. Introduction

Cardiovascular disease (CVD) is the leading cause of death worldwide, and myocardial hypertrophy, as a major pathological process, has been a longstanding challenge without effective interventions [1]. Cardiac hypertrophy represents an adaptive response of cardiomyocytes to pressure overload conditions such as hypertension or aortic stenosis, and it primarily manifests as cellular enlargement, fibrosis, and metabolic dysfunction. Persistence of this state may progress to arrhythmias, heart failure, or eventual sudden cardiac death [2]. Metabolic reprogramming of cardiomyocytes is associated with the development of cardiac hypertrophy. Metabolic pathways involving glucose, lipids, amino acids, ketone bodies, and others progressively reveal their indispensable roles in the pathophysiological process of cardiac hypertrophy [3].

Glutamine metabolism is a crucial metabolic pathway for maintaining cellular homeostasis. This process begins with glutaminase (GLS) converting glutamine deamidation into glutamate. Subsequently, glutamate is converted to α-ketoglutarate (α-KG) by glutamate dehydrogenase (GLUD) or aminotransferases (such as alanine or aspartate aminotransferases). α-KG then enters the tricarboxylic acid (TCA) cycle, serving as a key carbon source for energy production and biosynthesis [4] (Fig. 1). Glutamine initially gained attention primarily for its role in fueling the rapid growth of tumor cells, meeting their high-level synthetic demands and thereby promoting cell proliferation, migration, and invasion [5,6]. However, as research into glutamine metabolism deepens, its role in pathological cardiac hypertrophy has gradually been revealed. In the heart, glutamine metabolism exhibits unique characteristics. Studies indicate that the human heart actually secretes rather than consumes most amino acids, suggesting the presence of active proteolytic processes with degradation rates approximately ten times higher than in skeletal muscle. The heart specifically secretes glutamine and other nitrogen-rich amino acids while releasing intermediates of the TCA cycle, balancing carbon recovery from amino acid degradation. This discovery challenges conventional understanding of cardiac metabolism, suggesting glutamine metabolism plays a specialized role in maintaining cardiac energy homeostasis [7]. Most studies indicate that increased glutamine metabolism provides energy for cardiac cell hypertrophy, this regulatory mechanism is primarily achieved through modulation of key enzymes or transporters involved in glutamine metabolism [8]. As a consequence of the growing number of current studies on glutamine metabolism in cardiomyocyte hypertrophy, there is an urgent need to summarize how these metabolic alterations occur and whether they can provide novel therapeutic targets for pathological myocardial hypertrophy. Therefore, this paper focuses primarily on changes in glutamine metabolism and their molecular mechanisms during the process of pathological hypertrophy of cardiomyocytes, while also examining the interactions with lipid metabolism and glutamine supplementation strategies in cardiovascular diseases, with the aim of providing new insights for the treatment of cardiac hypertrophy and heart failure.

**Fig. 1.**
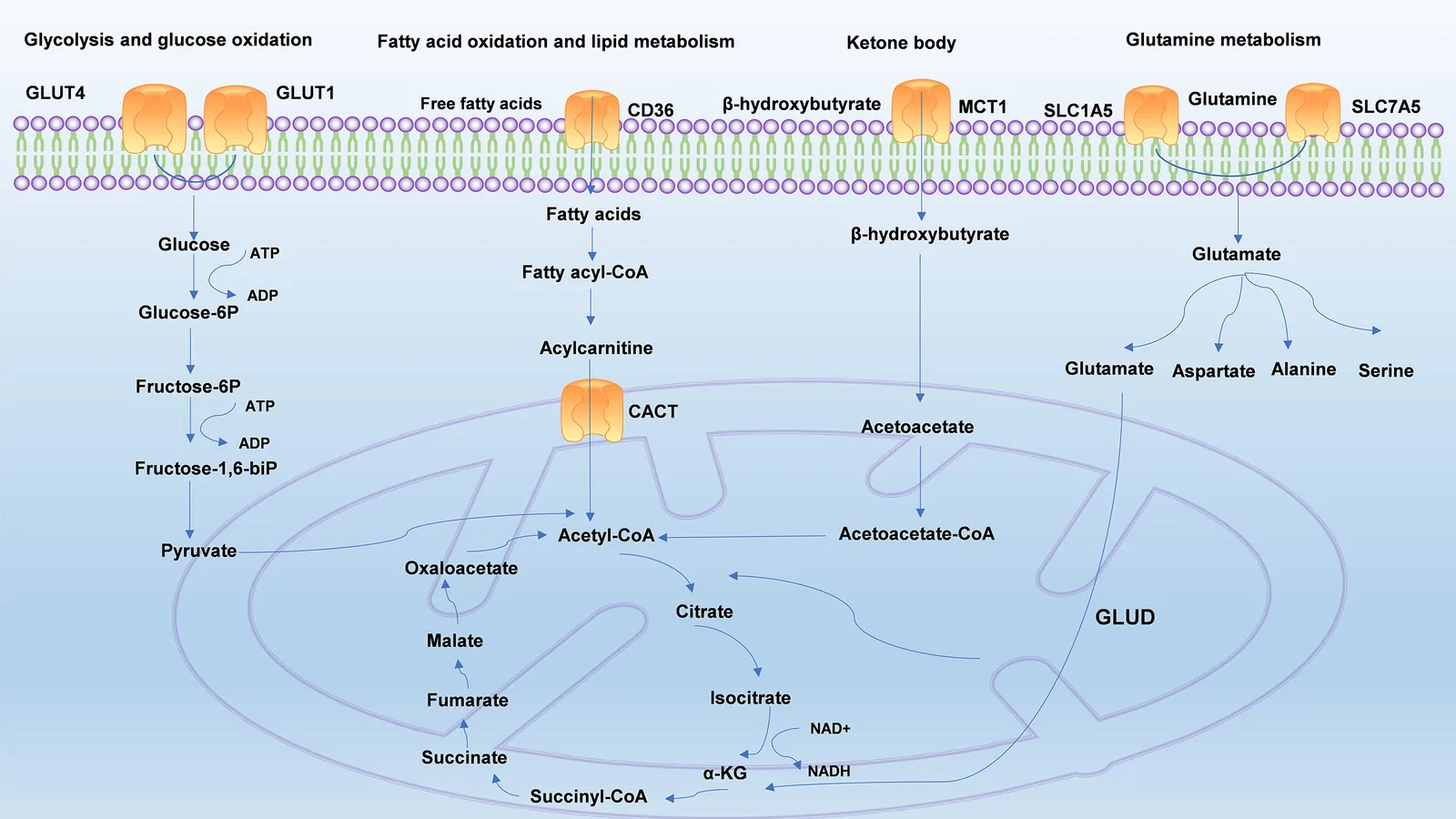
**Metabolism in cardiac myocytes**. Myocardial cells employ multiple interconnected energy metabolism pathways. Under aerobic conditions, glucose generates acetyl-CoA in mitochondria, which then produces adenosine triphosphate (ATP) via the tricarboxylic acid (TCA) cycle. Fatty acid oxidation and ketone utilization also yield acetyl-CoA. Meanwhile, glutamine metabolism contributes to the TCA cycle through α-KG recycling. GLUT1, Glucose Transporter Type 1; GLUT4, Glucose Transporter Type 4; α-KG, α-ketoglutarate; CACT, Carnitine-Acylcarnitine Translocase; MCT1, Monocarboxylate Transporter 1; SLC1A5, Solute Carrier Family 1 Member 5; SLC7A5, Solute Carrier Family 7 Member 5; GLUD, Glutamate dehydrogenase.

A literature search was conducted in the PubMed, Web of Science, and Scopus databases. Only original English-language articles and peer-reviewed review articles were included. Two authors independently screened titles/abstracts and full texts; any disagreements were resolved by consensus. Additional relevant literature was identified by manually searching the reference lists of the included articles.

## 2. Glutamine Metabolism in Physiological and Pathological Cardiomyocyte Hypertrophy

Glutamine metabolism refers to the conversion of glutamine into glutamate and free ammonia by glutaminase, and the conversion of glutamate into α-ketoglutarate by glutamate dehydrogenase. Subsequently, α-KG enters the TCA cycle to participate in glucose metabolism. Consequently, glutamine can serve as a carbon source supplementing the TCA cycle [9]. Additionally, glutamine can undergo complete oxidation to produce adenosine triphosphate (ATP), serving as a nitrogen source for the synthesis of nucleotides and other non-essential amino acids [10]. Simultaneously, its metabolic product glutamate serves as one of the raw materials for synthesizing glutathione. Together with NADPH, it functions as a reducing agent in maintaining intracellular redox homeostasis, which is beneficial for normal cell proliferation [11,12]. Overall, glutamine metabolism is a critically important metabolic pathway for cellular function and homeostasis. While it has been extensively studied in tumor cells, its regulatory role in the structural and functional aspects of cardiomyocyte hypertrophy requires a more detailed summary.

Physiological myocardial hypertrophy, such as that caused by regular exercise (athlete’s heart) or pregnancy, is an adaptive and reversible process [13]. Its core characteristics include enlarged myocardial cell volume and enhanced contractile function, accompanied by efficient metabolic remodeling to support this growth. Unlike the metabolic dysfunction and functional decline associated with pathological hypertrophy (such as that caused by hypertension or heart failure), the metabolic remodeling in physiological hypertrophy is coordinated, efficient, and energy-conserving [14,15]. Physiological hypertrophy is triggered by limited signals, including growth hormones (such as thyroid hormones, insulin, insulin-like growth factor 1, and vascular endothelial growth factor) and restricted intracellular signaling pathways (e.g., Phosphatidylinositol 3-kinase‌ [PI3K], Protein Kinase B [AKT], 5'-AMP-activated protein kinase‌ [AMPK], and mechanistic target of rapamycin [mTOR]) that influence gene transcription, protein translation, and metabolism [16]. Under physiological conditions, the primary energy source for the adult heart is fatty acid oxidation (accounting for approximately 60–90% of ATP production), while glucose oxidation contributes about 10–40% [17]. However, during physiological hypertrophy, the utilization and efficiency of glucose metabolism significantly increase to meet cellular biosynthetic demands and high-efficiency energy production. Glucose metabolism ensures adequate energy supply and provides biosynthetic carbon skeletons by enhancing glucose uptake, glycolysis, and glucose oxidation. Glutamine, as a crucial replenishing amino acid, enters the TCA cycle by converting into α-KG while simultaneously driving protein synthesis to maintain metabolic homeostasis. These two processes are tightly coupled through the TCA cycle, collectively forming the metabolic foundation supporting healthy cardiac hypertrophy [18,19].

However, in pathological myocardial hypertrophy, metabolic pathways also significantly change, leading to impaired myocardial energy supply and functional impairment [20]. Pathological myocardial hypertrophy is caused by persistent pressure overload (such as hypertension, aortic valve stenosis) or excessive neuroendocrine activation (such as angiotensin II) [21]. This is a maladaptive process that ultimately leads to heart failure. Its core characteristics are metabolic dysfunction and loss of metabolic flexibility, where cardiomyocytes fail to efficiently generate energy and synthesize new substances, resulting in functional decline. Unlike the comprehensive, coordinated enhancement seen in physiological hypertrophy, glucose metabolism in pathological hypertrophy is disordered and inefficient [3]. In glucose metabolism, increased glucose uptake is accompanied by diminished oxidative capacity. Upregulation of glycolytic enzyme expression and activity leads to a substantial increase in pyruvate production. Accumulated pyruvate is converted to lactate in the cytoplasm, causing intracellular acidosis that further impairs myocardial contractility [22]. Simultaneously, α-KG generated from glutamine metabolism directly enters the TCA cycle, attempting to replenish depleted TCA cycle intermediates caused by impaired glucose oxidation (PDH inhibition) and reduced fatty acid oxidation efficiency [23,24]. Some studies suggest that the hexosamine biosynthesis pathway (HBP) may be a potential candidate for glutamine’s effects on cardiac metabolism. Glutamine supplementation improved cardiac contractile function under conditions of restricted pyruvate replenishment, indicating that glutamine’s metabolic influence may extend beyond mere TCA cycle supplementation [25]. Unlike physiological myocardial hypertrophy, this represents a compensatory, desperate energy remedy—a hallmark of maladaptation. The heart attempts to use it to compensate for massive energy deficits and biosynthetic demands. Overall, whether in physiological or pathological myocardial hypertrophy, the occurrence of glutamine metabolic reprogramming is intertwined with carbohydrate metabolism, which complicates the study of glutamine metabolism.

## 3. The Dual Role of Glutamine Metabolism in Cardiomyocyte Hypertrophy

Glutamine, as the most abundant free amino acid in the body, serves not only as a building block for protein synthesis but also as an essential substrate for multiple biosynthetic pathways. In cardiomyocytes, glutamine metabolism provides carbon sources for the tricarboxylic acid cycle via the transglutaminase reaction, regulates redox status through glutathione synthesis, and influences multiple pathophysiological processes via signaling modulation. Notably, glutamine metabolism exhibits a distinct dual role in cardiomyocyte hypertrophy: it may exert protective effects by sustaining energy metabolism and reducing oxidative stress, while simultaneously contributing to hypertrophic processes by promoting anabolic metabolism and exacerbating metabolic remodeling.

## 4. The Protective Role of Glutamine Metabolism in Cardiomyocyte Hypertrophy

In pressure overload-induced cardiac hypertrophy, the heart undergoes significant metabolic remodeling characterized by reduced fatty acid oxidation, increased glucose utilization, and consumption of tricarboxylic acid cycle intermediates. Under these conditions, glutamine catabolism serves as a crucial source for compensatory reactions and is essential for maintaining cardiac function. First, glutamine is closely associated with cell proliferation. Human cardiomyocytes are known to have limited proliferative potential. However, mouse neonates and zebrafish can regenerate their hearts through cardiomyocyte dedifferentiation and proliferation. Zebrafish and neonatal mouse hearts exhibit elevated glutamine levels, making them susceptible to amino acid-driven activation of the mTOR signaling pathway. This activation is essential for *in vivo* regeneration in zebrafish cardiomyocytes [26]. Autologous cardiac progenitor cell (CPC) transplantation alleviates myocardial dysfunction in damaged hearts. Glutamine prolongs CPC doubling time and promotes survival under oxidative stress conditions, while enhancing CPC proliferation by improving mitochondrial function and stimulating mechanistic target of rapamycin complex 1 (mTORC1). Interventions that increase Gln uptake or oxidation may enhance CPC therapy [27].

Secondly, the protective effect of glutamine on cardiomyocytes is widely recognized to be achieved through replenishing glutathione or restoring TCA cycle function under oxidative stress conditions to maintain ATP supply. Glutamine serves as a crucial precursor for glutathione (GSH) synthesis. As one of the most important intracellular antioxidants, GSH helps eliminate excess reactive oxygen species (ROS) by enhancing its production. This glutamine metabolism pathway thereby mitigates oxidative damage and safeguards the structural and functional integrity of cardiomyocytes. Glutathione depletion induces cardiomyocyte hypertrophy, interstitial fibrosis, left ventricular enlargement, reduced ejection fraction, diminished myocardial contractility, impaired intracellular Ca^2+^ handling, impaired sarcoplasmic reticulum Ca^2+^ reuptake, and loss of mitochondrial integrity (mitochondrial swelling, mitochondrial dysfunction) [28]. Research indicates that combined administration of quercetin, glutamine, and alpha-tocopherol increases levels of interleukin-10 and glutathione while reducing the number of type I collagen fibers, thereby alleviating myocardial fibrosis in diabetic rats [29]. Oxidative stress is a prerequisite for cardiac hypertrophy. Studies indicate that under oxidative stress conditions, levels of glutamine, glutamate, and α-ketoglutarate in cardiomyocytes are significantly reduced, while glutaminase activity is upregulated. This indicates enhanced glutamine catabolism to compensate for the deficiency of tricarboxylic acid cycle intermediates, thereby exerting cardioprotective effects by maintaining ATP and GSH levels [30]. Therefore, from the perspective of supplementing glutathione and addressing oxidative stress, glutamine can suppress inflammatory responses and enhance cellular antioxidant defenses, thereby providing short-term protection for cardiomyocytes.

## 5. The Promoting Role of Glutamine Metabolism in Cardiomyocyte Hypertrophy

Although glutamine metabolism exerts protective effects under specific conditions, it may promote myocardial hypertrophy in other circumstances. Myocardial cell hypertrophy is fundamentally an anabolic process requiring substantial biosynthetic precursors to support protein synthesis, cell volume expansion, and organelle proliferation. As both an amino acid donor and carbon source, glutamine supports these synthetic processes through multiple mechanisms. First, α-KG produced from glutamine catabolism enters the tricarboxylic acid cycle, where it can be converted into citrate. Once transported into the cytoplasm, citrate provides acetyl-CoA for fatty acid synthesis, supporting the biosynthesis of membrane lipids. Second, the amino group of glutamine can be utilized for nucleotide synthesis and non-essential amino acid synthesis, meeting the demands of nucleic acid and protein synthesis in hypertrophied cardiomyocytes. Metabolic alterations in hypertrophic cardiomyocytes were revealed in the transverse aortic constriction (TAC) model in animals, where TAC is accompanied by significant increases in glycolysis, mitochondrial oxidative metabolism, glucose metabolism to replenish substrates, and glutamine metabolism [31].

It is well known that heart failure is associated with myocardial fibrosis. The persistent presence of myofibroblasts in chronic heart failure leads to progressive cardiac dysfunction and maladaptive remodeling. Enhanced glutamine catabolism mediates increased α-KG abundance. Inhibition of glutamine catabolism prevents myofibroblast formation, manifested by reduced αSMA+ cells, collagen gel contraction, collagen abundance, and bioenergetic responses, while also preventing TGFβ-mediated histone demethylation [32]. Some scholars have proposed that enhanced glutamine dissolution and increased bioavailability of α-KG may facilitate changes in fibrosis and cellular gene expression. This alteration in glutamine is mediated by glutaminase 1 (GLS1) [33]. Recent research findings indicate that glutamine dissolution serves as a key mediator sustaining myofibroblasts in mouse heart failure models and those directly isolated from diseased human hearts. This occurs because glutamine, as a critical carbon source for metabolic processes, protein biosynthesis, and epigenetic regulation of gene expression, facilitates myofibroblast differentiation and the fibrotic phenotype [34]. Concurrently, clinical studies have evaluated the association between glutamine metabolism and the incidence of heart failure and atrial fibrillation in individuals at high risk for cardiovascular disease. Results suggest that elevated plasma glutamate levels and a lower glutamine-to-glutamate ratio may increase the risk of heart failure, but do not elevate the risk of atrial fibrillation [35].

Many cancer cells exhibit “glutamine addiction”, meaning they consume large amounts of glutamine to support their rapid proliferation. This glutamine not only provides energy and biosynthetic precursors via the TCA cycle, but a significant portion is also “hijacked” by glutamine-fructose-6-phosphate amidotransferase 1 (GFAT1) into the HBP [36]. During cardiac hypertrophy, intracellular glucose may also be diverted to alternative metabolic pathways. GFAT1 serves as the key entry point for glutamine into the HBP. Hypertrophic growth in cardiomyocytes induces the HBP, leading to increased utilization of glutamine and glucose for uridine diphosphate N-acetylglucosamine (UDP-GlcNAc) production. Notably, GFAT1 inhibition markedly suppresses epinephrine-induced hypertrophic growth, potentially offering a novel therapeutic perspective for heart failure [37]. Cardiac hypertrophy is frequently accompanied by metabolic reprogramming, characterized by alterations in substrate preferences and metabolic pathways. Changes in glutamine metabolism may constitute a significant component of this reprogramming. Collectively, these studies suggest that prolonged activation of glutamine catabolism may lead to metabolic accumulation and signaling pathway abnormalities, promoting myocardial fibrosis and hypertrophy. However, this process is typically chronic and sustained, differing from the early protective effects observed.

In summary, based on current research evidence, it remains unclear under what conditions glutamine metabolism exerts a protective effect and under what conditions it promotes myocardial hypertrophy. Therefore, whether glutamine metabolism plays a beneficial, harmful, or neutral role in the process of myocardial hypertrophy remains an open question.

## 6. How Does Glutamine Metabolism Regulate Cardiac Hypertrophy?

### 6.1 Glutamine Metabolism Enzymes

Key enzymes in glutamine metabolism are tightly regulated at multiple levels, with these controls determining the direction and intensity of metabolic flux. GLS serves as the rate-limiting enzyme for glutamine degradation, and its expression and activity are subject to multifaceted regulation through transcriptional control, post-translational modifications, and allosteric modulation. Under oxidative stress conditions, GLS activity is upregulated in cardiomyocytes, promoting glutamine breakdown. Inhibition of GLS1 reduces the production of glutamine-derived aspartate and citrate, which are essential for nucleic acid and lipid biosynthesis and may contribute to the suppression of cardiac hypertrophy and fibrosis [23]. Additionally, studies have revealed that the long non-coding RNA Snhg7 induces ferroptosis by interacting with the cardiac transcription factor T-box transcription factor 5 (Tbx5). Furthermore, the glutaminase 2 (GLS2) promoter binds to Tbx5, thereby regulating ferroptosis activity in hypertrophic cardiomyocytes [38].

GLUD is an enzyme that catalyzes the conversion of glutamate to α-KG. It participates in isoproterenol (ISO)-induced cardiac hypertrophy by activating the mammalian target of rapamycin (mTOR). Research confirms that GDH silencing protects rats from ISO-induced cardiac hypertrophy, indicating it is a potential target for myocardial cell hypertrophy [39]. Ginsenoside Rg1 protects cardiomyocytes from H/R-induced oxidative stress damage by maintaining mitochondrial dynamics through GDH regulation [40].

Transaminases such as aspartate transaminase (AST/GOT) and alanine transaminase (ALT/GPT). They transfer the amino group from glutamic acid to other keto acids, producing the corresponding amino acids and α-KG. This represents another pathway for generating α-KG, while simultaneously yielding other non-essential amino acids (aspartic acid, alanine) for nucleotide and protein synthesis. GOT mRNA expression, activity, and protein levels in juvenile rat hearts increase with age-related physiological myocardial hypertrophy [41]. In TAC mice and hypertrophied cardiomyocytes, mitochondrial uncoupling protein 3 (UCP3) alleviates cardiac hypertrophy by inhibiting aspartate aminotransferase [42].

Glutamine synthetase is the sole enzyme responsible for endogenous glutamine synthesis. It eliminates toxic ammonia within cells and maintains intracellular glutamine concentrations. Although expressed by endothelial cells, their glutamine synthesis activity is negligible under physiological glutamine levels. Glutamine synthetase undergoes palmitoylation and interacts with RhoJ (Rho-related GTP-binding protein) to maintain RhoJ palmitoylation, thereby inhibiting endothelial cell migration in pathological angiogenesis [43]. Research has confirmed that cardiac cell proliferation in zebrafish is promoted by glutamine synthetase and regulated by carnitine palmitoyltransferase 1b (Cpt1b), providing new insights into the importance of fatty acid metabolism in cardiac development and the interactions between fatty acid and amino acid metabolic pathways [44].

Glutamine: Fructose-6-phosphate amidotransferase (GFAT) is the rate-limiting enzyme in the HBP. This pathway consumes glutamine and glucose, with the end product UDP-GlcNAc serving as a substrate for O-GlcNAc glycosylation modification of proteins. This modification constitutes a crucial signaling regulatory mechanism closely associated with diabetic complications, cancer, and neurodegenerative diseases [45]. GFAT has been demonstrated to increase its expression in response to several hypertrophic stimuli, including ISO, and may represent a key therapeutic target for myocardial hypertrophy [46]. AMPK has been shown to inhibit cardiac hypertrophy primarily by suppressing O-GlcNAcylation through controlling GFAT phosphorylation, blocking cardiomyocyte hypertrophy [47]. Therefore, GFAT serves as a pivotal hub linking glutamine metabolism and the hexosamine biosynthetic pathway.

It is worth noting that another similarly named enzyme, transglutaminase (TGase), does not directly regulate glutamine metabolism. However, it indirectly influences glutamine metabolic pathways by consuming glutamine [48]. It catalyzes the reaction between the γ-amide group of a glutamine residue (from one protein) and the ε-amino group of a lysine residue (from another protein), forming a covalent bond (an isopeptide bond) that cross-links the two protein molecules together. In this process, the amide group of glutamine is consumed, and ammonia is released. It catalyzes the hydrolysis of glutamine, producing glutamic acid and ammonia. This is the first and most crucial step in cellular utilization of glutamine. The resulting glutamic acid can enter the tricarboxylic acid (TCA) cycle for energy production, serve as a neurotransmitter, or be used to synthesize other amino acids (such as proline) and antioxidants (like glutathione) [49,50,51]. Furthermore, studies have reported that transglutaminase regulates myocardial fibrosis and stress-induced cardiac hypertrophy [52]. The loss of transglutaminase is associated with myocardial hypertrophy, reduced collagen cross-linking, and enhanced matrix metalloproteinase (MMP)-2 in stress-overloaded myocardium. Upon binding to the matrix, recombinant tTG induces the synthesis of tissue inhibitor of metalloproteinases (TIMP)-1 through a transaminase-independent mechanism [53]. Transglutaminase 2 plays a crucial role in regulating myocyte contractility by promoting fatty acid metabolism, and offers a novel therapeutic target for preventing high-load cardiac contractile dysfunction [54]. Although transglutaminase does not primarily function in metabolism, its activity does influence intracellular glutamine levels. Since the transglutaminase reaction consumes glutamine and produces ammonia, it does indirectly affect intracellular glutamine levels and the equilibrium of associated metabolic networks.

### 6.2 Glutamine Transporter

Glutamine requires specific membrane transporters to enter cells; these transporters belong to the solute carrier (SLC) superfamily [55]. It is widely recognized that under stress conditions in heart failure, cardiomyocytes may increase glutamine uptake by upregulating the expression or activity of certain glutamine transporters. Solute Carrier Family 1 Member 5 (SLC1A5) is a neutral amino acid transporter critical for cellular glutamine homeostasis. Compared to healthy controls, SLC1A5 mRNA levels are suppressed in human hyperlipidemic samples, while SLC1A5 protein expression is also reduced in human failing cardiomyocytes. This leads to disrupted glutamine homeostasis and impaired myocardial energy balance [56]. SLC1A5 rarely acts alone. It frequently works in concert with another transporter called SLC7A5 (forming a heterodimer with SLC3A2) [57].

Increased glutamine metabolism has also been demonstrated in patients with right ventricular hypertrophy. In patients with pulmonary embolism-induced right heart failure, expression of glutamine transporters (SLC1A5 and SLC7A5) is significantly elevated [58]. Recent studies have revealed that epigenetic mechanisms may participate in regulating the expression of glutamine metabolism-related transporters. The E3 ubiquitin ligase tripartite motif-containing protein 35 (TRIM35) interacts with SLC7A5 in cardiac fibroblasts from fibrotic hearts of both mice and humans, thereby enhancing amino acid transport and activating the mTORC1 signaling pathway [59]. The regulation of glutamine metabolism extends beyond intake, with its efflux from cells also playing a significant role. For instance, the SLC7A5/SLC3A2 complex mediates glutamine efflux while simultaneously transporting essential amino acids like leucine into cells. This “exchange” mechanism is crucial for cellular perception of nutritional status and regulation of signaling pathways such as mTOR (Fig. 2).

**Fig. 2.**
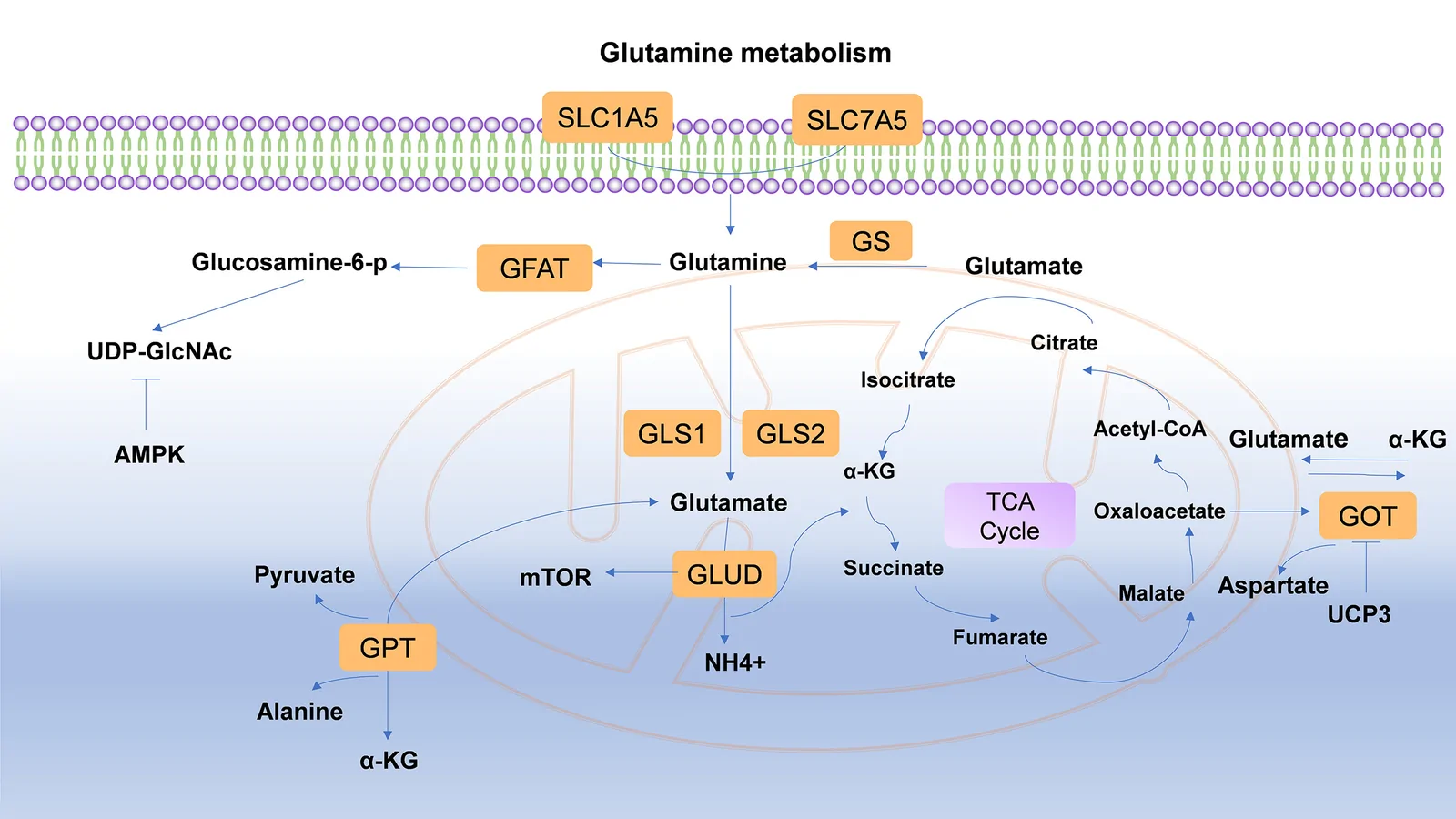
**Regulation of glutamine metabolism in cardiac myocyte hypertrophy**. Current understanding of glutamine metabolism regulation in cardiac hypertrophy primarily focuses on key enzymes and major transporters involved in glutamine metabolism. Both enzyme activity and protein levels can either promote or inhibit metabolic pathways. Glutamine metabolism plays a complex dual role in cardiomyocyte hypertrophy: it may exert protective effects by maintaining energy metabolism, enhancing antioxidant defenses, and suppressing inflammation, while simultaneously exacerbating hypertrophy by promoting metabolic remodeling and inducing mitochondrial dysfunction. This duality depends on multiple factors including disease type, metabolic environment, stress intensity, and signaling pathway activation status. SLC1A5, Solute Carrier Family 1 Member 5; SLC7A5, Solute Carrier Family 7 Member 5; GFAT, Fructose-6-phosphate Amidotransferase; GS, Glutamine Synthetase; GLS1, Glutaminase 1; GLS2, Glutaminase 2; GLUD, Glutamate Dehydrogenase; GPT, Alanine Aminotransferase; GOT, Aspartate Aminotransferase; UCP3, Mitochondrial Uncoupling Protein 3; AMPK, AMP-activated protein kinase; mTOR, mechanistic target of rapamycin.

Overall, existing research on glutamine metabolism regulation in cardiomyocyte hypertrophy primarily focuses on key glutamine metabolic enzymes and major transporters SLC1A5 and SLC7A5. Studies involving other mechanisms such as epigenetic regulation, histones, lactation, and non-coding RNAs remain scarce and require further investigation for confirmation (Table 1, Ref. [23,38,39,41,42,44,46,47,56,58,59]).

**Table 1.** **Glutamine metabolism regulates cardiac myocyte hypertrophy pathways**.

		Model	Mechanism	Consequence	References
Glutamine Metabolism Enzymes					
	GLS1	Animal	Regulating aspartic acid and citrate	Inhibition of GLS1 reduces cardiac hypertrophy and myocardial fibrosis	[23]
	GLS2	Animal	Regulates ferroptosis in cardiomyocytes	GLS2 promoter binding to Tbx5 exacerbates myocardial hypertrophy	[38]
	GLUD	Animal	Activates mTOR; regulates mitochondria	Activation of GLUD aggravates myocardial hypertrophy	[39]
	AST/GOT	Animal	UCP3 can inhibit aspartate aminotransferase	Inhibition of aspartate aminotransferase reduces myocardial cell hypertrophy	[41,42]
	Glutamine synthetase	Animal	CPT1b regulates glutamine synthetase	Promotes myocardial cell proliferation	[44]
	GFAT	Animal	AMPK regulates GFAT phosphorylation to inhibit O-GlcNAcylation	Inhibits myocardial cell hypertrophy	[46,47]
Glutamine Transporter					
	SLC1A5	Animal	Increased protein expression promotes transport	Exacerbates myocardial hypertrophy	[56,58]
	SLC7A5	Animal	Increased protein expression promotes transport	Exacerbates myocardial hypertrophy	[58,59]

### 6.3 Interactions Between Glutamine Metabolism and Lipid Metabolism

In cardiac energy metabolism, fatty acid oxidation serves as the primary source of ATP, accounting for approximately 50–70% of energy supply in healthy adult hearts. The substantial acetyl-CoA generated through fatty acid β-oxidation enters the TCA cycle, further producing ATP via oxidative phosphorylation. In failing hearts, fatty acid oxidation diminishes while ketone and lactate utilization increases, accompanied by elevated proteolysis rates. This metabolic reprogramming partially compensates for the energy deficit caused by reduced fatty acid oxidation but may also lead to net myocardial protein loss and impaired function. This process involves multi-level interactions with glutamine metabolism [7].

The mTOR signaling pathway senses amino acid availability and regulates lipid metabolism. Metabolically remodeled hearts exhibit impaired fatty acid oxidation, reducing their capacity to generate energy from fatty acids. mTORC1 is activated under stress overload, β-adrenergic stimulation, and hypertrophic signaling from angiotensin II and IGF-1. Excessive mTORC1 activation inhibits cardiac fatty acid oxidation, serving as a prerequisite for developing and maintaining compensatory myocardial hypertrophy [60]. It has been confirmed that many tumor cells are highly dependent on glutamine, and their glutamine metabolic reprogramming can continuously activate mTORC1, driving tumor proliferation and survival [61]. The metabolic process of glutamine contributes to the activation of Rag GTPases. Activated Rag heterodimers recruit mTORC1 to the lysosomal surface, a critical step in its activation [62,63,64]. Simultaneously, glutamine metabolism exerts positive regulation on mTOR. mTORC1 can upregulate the expression or activity of glutaminase, the rate-limiting enzyme in glutamine degradation. Increased GLS activity promotes the catabolic metabolism of glutamine [65,66]. Overall, the mTOR pathway serves as a provider of fatty acid carbon sources in hypertrophic cardiomyocytes by sensing glutamine availability and coordinating its metabolism with fatty acid synthesis.

### 6.4 Glutamine Supplementation Strategies in Cardiovascular Diseases

Considering that glutamine is the most abundant free amino acid in the human body, it not only plays a critical role in protein synthesis but also participates in regulating various biological processes. Recent studies have revealed that glutamine serves not only as a vital energy source for cell proliferation but also functions as a signaling molecule, directly regulating intracellular metabolism with hormone-like effects [67]. Supplementing glutamine or its metabolites under specific pathological conditions may be beneficial. Notably, as one of the primary energy sources for macrophages, alterations in glutamine metabolism directly influence macrophage phenotypic switching, functional execution, and adaptive responses in cardiovascular diseases [68,69].

The role of glutamine in regulating cardiac remodeling after myocardial infarction has also been uncovered. Studies indicate that glutamine metabolism is altered in both the distal remodeling region and the infarct area infiltrated by macrophages following myocardial infarction. Supplementing glutamine improves left ventricular function, which is associated with regulating glutamine metabolic reprogramming in myocardial macrophages [70]. Simultaneously, glutamine supplementation provides protective effects for cardiac surgery patients, particularly those undergoing cardiopulmonary bypass. Coronary artery bypass grafting (CABG) under cardiopulmonary bypass (CPB) and aortic cross-clamping (AoX) induces myocardial ischemia. Studies indicate that glutamine supplementation protects cardiomyocytes during myocardial ischemia, as evidenced by reduced levels of troponin I [71]. For patients undergoing CABG with low ejection fraction, perioperative administration of 0.5 g/kg glutamine solution provides myocardial protection [72].

Heart failure represents the terminal stage of various cardiac diseases, including myocardial hypertrophy, characterized by the heart’s pumping capacity failing to meet metabolic demands. The protective role of glutamine in heart failure primarily manifests through its regulation of cardiac fibrosis. Cardiac fibrosis, a key pathological basis of heart failure, is characterized by excessive extracellular matrix deposition and impaired cardiac diastolic function. Co-administration of quercetin, glutamine, and α-tocopherol increased circulating interleukin-10 (IL-10) and glutathione levels while reducing the number of type I collagen fibers [29]. L-glutamine has been shown to alleviate cardiac metabolic stress. Fructose-rich beverages during pregnancy induce cardiac hypertrophy accompanied by increased pyruvate dehydrogenase kinase 4 (PDK4). Supplementation with L-glutamine inhibits PDK4 activity, thereby preventing the progression of cardiac hypertrophy. This suggests glutamine may represent a novel therapeutic intervention for cardiac hypertrophy, particularly during pregnancy [73].

Glutamine, an amino acid with multi-target protective effects, demonstrates significant cardioprotective benefits across various cardiac disease models. Its mechanisms involve multiple aspects including energy metabolism regulation, oxidative stress reduction, inflammatory response modulation, cellular protection mechanisms, and signaling pathway control. By promoting the expression of protective proteins, improving myocardial energy metabolism, and suppressing excessive inflammatory responses and apoptosis, glutamine helps maintain cardiac structural and functional integrity [67,74,75].

## 7. Limitation

Several limitations of this review should be acknowledged. First, there remain important evidence gaps that limit the strength of our conclusions. Nearly all studies discussed are preclinical (*in vitro* or animal models), and no clinical data exist on glutamine metabolism in human cardiac hypertrophy. Therefore, any reference to “therapeutic potential” should be considered as hypothesis‑generating rather than clinically established. Second, the field currently lacks sufficient data to define the boundary conditions under which glutamine metabolism exerts apparently opposing effects (protective versus pro‑hypertrophic roles). The available studies use different cell types, stimuli, doses, and time points, and no systematic *in vivo* investigation has determined when or how a switch might occur. Third, the discussion of crosstalk between glutamine and other metabolic pathways (glucose, fatty acids) is limited largely to the mTORC1 axis, because direct mechanistic studies in hypertrophic cardiomyocytes are sparse. We have noted this as a major gap that requires future investigation. Finally, while we have attempted to integrate findings and highlight controversies, the rapidly evolving nature of this field means that some inconsistencies remain unresolved. We have pointed out contradictory results where they exist and have proposed testable hypotheses to resolve them, but continuing research will be necessary to reach definitive conclusions. Despite these limitations, we believe this review provides a structured and critical synthesis of current knowledge on glutamine metabolism in cardiac hypertrophy and identifies priority areas for future translational studies.

## 8. Conclusion and Prospects

Despite significant advances in understanding glutamine metabolism’s role in cardiac hypertrophy, several questions remain to be explored: First, it is necessary to clarify the dynamic changes and specific functions of glutamine metabolism during different stages of cardiac hypertrophy. Current research predominantly focuses on specific time points within the disease process, lacking continuous dynamic observation. Utilizing metabolomics to track changes in glutamine metabolic flux may provide a more comprehensive understanding of its role in cardiac hypertrophy [76,77]. Second, the interactions between glutamine metabolism and other metabolic pathways (such as glucose metabolism and fatty acid metabolism) require in-depth analysis [78,79]. Cardiac structural remodeling inevitably leads to cardiac metabolic remodeling, which involves alterations in multiple metabolic pathways. These changes are not isolated but interconnected and mutually influencing [3,80,81,82]. Investigating how glutamine metabolism interacts with other metabolic pathways may provide a more comprehensive understanding of the metabolic mechanisms underlying cardiac hypertrophy. Third, more specific intervention tools need to be developed. Current studies predominantly employ broad-spectrum inhibitors or supplements, lacking tissue specificity and target specificity. Constructing cell-specific knockout animal models using gene editing techniques or developing tissue-specific targeted drugs may enable more precise elucidation of glutamine metabolism’s role [83,84,85]. Finally, clinical translational research must be strengthened. Currently, most studies remain confined to the basic research stage. Caution should be exercised when extrapolating findings from studies involving fibroblasts, macrophages, or regenerative models to cardiomyocyte hypertrophy. More clinical studies are needed to validate the role of glutamine metabolism in human cardiac hypertrophy and explore its potential as a diagnostic biomarker or therapeutic target.

Glutamine metabolism plays a complex dual role in cardiomyocyte hypertrophy. It may exert protective effects by maintaining energy metabolism, enhancing antioxidant defenses, and suppressing inflammation, while simultaneously exacerbating myocardial hypertrophy by supplying biosynthetic precursors, promoting metabolic reprogramming, and inducing mitochondrial dysfunction. This duality depends on multiple factors including disease type, metabolic environment, stress intensity, and signaling pathway activation status. Future research requires a deeper elucidation of the specific patterns of glutamine metabolism changes in different types of myocardial hypertrophy (such as pressure overload, ischemic, and inflammatory), along with the identification of its upstream regulatory mechanisms and downstream effector molecules. Simultaneously, developing specific modulators targeting key enzymes in glutamine metabolism and exploring personalized therapeutic strategies represent important research directions. Furthermore, the interactions between glutamine metabolism and other metabolic processes in cardiac hypertrophy remain unclear, advancements in metabolomics and molecular imaging technologies will enable real-time monitoring of myocardial glutamine metabolism, providing a foundation for precision medicine. Ultimately, comprehensive regulation of the glutamine metabolic network—rather than targeting individual enzymes or pathways alone—may offer more promising clinical applications for the prevention and treatment of cardiac hypertrophy.

In summary, glutamine metabolism, as a crucial component of myocardial cell metabolic reprogramming, offers valuable insights into the pathophysiological mechanisms of cardiac hypertrophy and the development of novel therapeutic strategies through its dual roles. By delicately modulating this metabolic pathway, we may achieve more effective prevention and treatment of cardiac hypertrophy in the future.
